# Syneresis investigations of lacto-fermented sodium caseinate in a mixed model system

**DOI:** 10.1186/s12896-019-0539-1

**Published:** 2019-08-02

**Authors:** Soumaya El Bouchikhi, Philippe Pagès, Yassir El Alaoui, Azeddine Ibrahimi, Yahya Bensouda

**Affiliations:** 1 Laboratory of Pharmaceutics, Faculty of Medicine and Pharmacy, Mohammed V University, Rabat, Morocco; 2 Laboratory of Medical Biotechnology, Faculty of Medicine and Pharmacy, Mohammed the Vth University in Rabat, Rabat, Morocco; 3 PhP Stats, Création et analyse d’information, Conseil, études et formations en statistique, 19, rue Pasteur, 94170 Le Perreux, France

**Keywords:** Syneresis, Sodium caseinate, Lacto-fermentation, Starch, Acid gel, Non-dairy product

## Abstract

**Background:**

The textural characteristics of fermented dairy products are important quality parameters that play a major role in their stability and consumer’s acceptance. The aim of this study was to investigate the influence of sodium caseinate, starch, lactose and lactic acid bacteria as ferment on the syneresis in a mixed model system, and to evaluate their impact on the acid gel formation throughout pH and zeta potential monitoring. Accordingly, a protocol was designed to perform an experimental design by using a mixture of the selected factors**.**

**Results:**

A significant decrease of syneresis was detected in all mixtures at 8% of sodium caseinate, ranging between a minimum of 1.8% and a maximum of 20.6% compared to the mixtures at 3% of sodium caseinate in which the syneresis decrease had ranged between a minimum of 22.2% and a maximum of 47.8%. The addition of starch had a significant impact on the acidification profile and on the syneresis of the fermented mixed model. Moreover, the monitoring of pH and zeta potential during the lacto-fermentation process has also led to a better understanding of the acid gelation and the syneresis variations.

**Conclusion:**

Syneresis varies very closely with sodium caseinate concentration, starch concentration and also with their association, regardless of the concentrations of lactose and ferment. In fact syneresis could be reduced to an optimum level if a sodium caseinate-starch mixed system is employed: Less syneresis gels could be obtained at a sodium caseinate concentration above 5% if starch is used above 1%**.**

## Background

Fermented food products have a great nutritional value and beneficial effect on host gut micro-biome due to their probiotics bacteria, which have a positive impact on human health [1]. In fact Gut micro-biome can directly influence host wellbeing by providing nutritional, metabolic and immunological benefits [2]. In the market, there is a growth in the development of fermented functional dairy products reflecting great interest in studying dairy products that demonstrate health benefits. This market expansion is highly attributed to the food technologist’s research, in particular, the development of new ingredients as flavors, fat substitutes, and protein ingredients to be added in the product in order to improve different qualitative and technical aspects. However more innovative efforts are needed to develop new internal matrix from a variety of substrates, and evaluate its fermentation. On the basis of recent developments, it is anticipated that fermented functional products will continue to be a significant component within the functional food market [3].

Milk, is a natural source of human nutrition and functional food materials that serves as the raw material for the production of dairy products, but also used as foodstuffs in their own right and as ingredients in the manufacture of formulated and processed foods: proteins, lactose, water-soluble vitamins and minerals. Milk proteins can be divided into two species: casein and whey proteins. The beneficial health properties resulting from the consumption of fermented milks have been known for many years. Fermented dairy products are usually made from standardized milk with fat content of 0–3.5% and non fat milk solid content of 11–13% through acidification with lactic acid bacteria. Among the different process parameters such as heat treatment, fermentation, storage conditions and starter culture, milk base composition has a big impact on the gel structure formation.

To improve and optimize the properties and the nutritional qualities of fermented dairy and non-dairy products, a variety of ingredients are used on both the process and the composition. It was also reported that casein-based products tended to produce firmer gels with less syneresis than yogurts fortified with whey protein [4]. Damin et al. (2009) showed that yogurt made with milk supplemented with sodium caseinate resulted in significant increase in firmness. The increase in milk solids may be achieved by the addition of skimmed milk powder or through the concentration of a milk portion by ultrafiltration. In a previous study the influence of milk protein-based ingredients on the textural characteristics, the sensory properties, and the microstructure of probiotic yogurt during a refrigerated storage period of 28 days was studied; and it was reported that probiotic yogurt from milk fortified with sodium caseinate showed much higher values of firmness and viscosities than yogurt from skimmed milk powder or whey protein concentrate fortified milk [5]. Heat treatment also promotes chemical changes, which improve the milk solids as a fermentation substrate by inducing whey protein-casein interaction through *β*-lacto-globulin-*κ*-casein interactions, which improve the texture of the product and reduce syneresis [6]. Conventionally, syneresis is considered as a major defect in products that are based on acid-induced gels [7]. Consumers perceive the separation of whey (syneresis) from yogurt during shelf life as a defect, which is a major subject of concern in the dairy industry [8]. Therefore, The textural characteristics of fermented dairy products are considered as important quality parameters that play an important role in their stability and their consumer’s acceptance. Moreover, additives such as pectin, guar gum, cornstarch and gelatin may be used to stabilize the product against syneresis, which also contributes to texture and viscosity.

The matrix or the gel formation is a key property in the acid induced gel formulation, in which casein plays a major role due to its structure properties [5]. It is also known that these gels consist of a particulate network of casein particles linked together to trap water and hold it in place [9, 10]. The main factors governing the formation of acid casein gels are casein concentration, pH, temperature and ionic strength [11, 12]. Before acidification the individual molecules of casein interact with each other and form associate structures called micelles, which are stabilized by electrostatic and steric interactions [4]. During fermentation, lactic acid bacteria use lactose to produce lactic acid that neutralizes gradually the negative charges on the surface of the casein micelles. This causes a decrease of the repulsive interactions and the onset of big aggregates, leading to a destabilization of the dispersion as soon as the pH drops below 5 [13]. The reduction in the negative charge on caseins occurs with the approach of the isoelectric point, which facilitates aggregation via electrostatic attraction between oppositely charged segments on the caseins as well as by enhanced hydrophobic interactions [14]. Electrostatic repulsion on the caseins decreases with the approach of the isoelectric point; in unheated milk this causes a collapse of the k-casein hairs (brush) onto the micelles surface (in heated milk the k-casein hairs are associated with denatured whey proteins and so do not collapse onto the micelle surface). The importance of both electrostatic and hydrophobic interactions for acid gelation was elegantly demonstrated by the cold acidification method developed by Roefs and van Vliet (1990) [15, 16]. At the isoelectric point of casein (pH 4.65), micelles lose completely their structure because of the whole dissolution of calcium’s micelles and the entire denaturation of casein, forming an acid gel protein-network [17, 18]. It was also reported that starch addition increases the value of the rheological and physical properties of yoghurts by improving consistency, increasing viscosity and reducing syneresis [19], which contributes to the formation of more stable dispersed acidified milk gelled systems [20, 21]. The nature of interaction between milk proteins and polysaccharides used as stabilizers depends on many factors including the specific types of intermolecular forces between them, their concentrations and environmental factors such as pH, ionic strength and calcium content [11]. For exemple the interactions of modified starches and casein included electrostatic adhesion, steric stabilization and hydrogen bond [22]. Therefore, a comprehensive study integrating convenient combinations of the main factors involved in the acid gel formation is essential for a closer understanding of syneresis mechanism through the proposed mixed model system.

The purpose of this study was to use sodium-caseinate as the main substrate to create physical building blocks within the continuous phase that may substitute the functionality of the removed milk ingredients and to produce an acid gel-network capable of holding the serum phase with an optimal stability. Accordingly, an experimental design was proposed by using a mixture design of the following selected factors: sodium caseinate, starch, lactose and lactic acid bacteria to determine the effect of these formulations on the syneresis of the formed acid gel protein network. The effects of these formulation variables on syneresis were evaluated through pH and zeta potential monitoring. Fermentation was performed with lactic acid bacteria *Lactobacillus delbrueckii* ssp. *Bulgaricus* combined to *streptococcus thermophilus* [23, 24]. This work provided more insight on how to reduce syneresis in fermented non-milk based products, which could be a promising venue in their development.

## Conclusion

This study demonstrated that sodium caseinate considerably decreased syneresis in the fermented model system compared to starch while ferment and lactose did not affect significantly syneresis. Moreover, the effect of starch on the acidification profile was revealed as an important element to consider in the understanding of the impact of reduced fermentation time on syneresis. It could be concluded from our trials that the optimum syneresis was achieved when the model bases were sodium caseinate and starch. Based on our results, we also showed that syneresis in the proposed model gel system could be reduced to an optimum level if a sodium caseinate-starch mixed system was employed: Less syneresis gels could be obtained at a sodium caseinate concentration above 5% if starch was used above 1%. Therefore, it will be worthwhile to make an exhaustive study integrating the most convenient combination of the selected factors and different steps of yogurt like manufacturing.

## Materials and methods

### Ingredients

The main ingredients used in this study were: Sodium caseinate (provided by Trade Bio-Industries Morocco); Native starch (Maïzena trade name, purchased from the local market); Lactose (LOBAL Chemie Laboratory) and Lactic acid bacteria: commercial yogurt starter culture freeze-dried containing *Strep. Thermophilus* and *Lactobacillus delbrueckii ssp. Bulgaricus* (provided by Trade Bio-Industries Morocco)*.* Freshly distilled water was used for preparation of all samples.

### Equipment

The main equipment used in this study was: Water bath set at 42 °C (Memmert WNE14); Fine test sieve (mesh width 0.25 mm); pH meter (Inlab sensor dairy Electrode. Mettler Toledo); Nanosizer ZS analyzer (Malvern version 6.2 serial number MAL1043134) and Design Expert 10 software edited by Stat-Ease inc.

### Syneresis monitoring

The ranges of the studied factors proposed for the determination of functional design space, showed in Table 1, were determined according to the concentrations range of sodium caseinate, starch, lactose and lactic acid bacteria used in fermented dairy products manufacturers [17] and designated as factors A, B, C and D respectively.

**Table 1:** Lower and upper limits of components used to make the experimental design

Factors	Lower limit	Upper limit	
Sodium caseinate (A)	3%	8%	
Starch (B)	0%	2%	
Lactose (C)	4%	9%	
Ferment (D)	0.2 g/l	0.5 g/l	

### Main experimental design

To define the formulation space for sodium-caseinate mixtures, a Complete Factorial Plan performed with Design Expert was selected to evaluate and to model the effects of sodium caseinate, Lactose, ferment and starch on decreasing the syneresis of the fermented product. This provides maximum information from a limited number of experiments as indicated in Table 2.

**Table 2:** The main experimental design for the selected factors. The completed plan is a “Complete Factorial Plan” with 4 Factors in 2 levels and 16 trials plus 3 points in the center. This plan made it possible to calculate the following and perform a Curvature test: The 4 Main Effects, the 6 Interactions of order 2, the 5 Interactions of order 3, the only Interaction of order 4

Sample	Space type	Sodium caseinate %(A)	Starch %(B)	Lactose %(C)	Ferment lg/l(D)	Dry matter %
1	Factorial	3	0	4	0.2	7
2	Factorial	3	0	4	0.5	7
3	Factorial	3	0	9	0.2	12
4	Factorial	3	0	9	0.5	12
5	Factorial	3	2	4	0.2	9
6	Factorial	3	2	4	0.5	9
7	Factorial	3	2	9	0.2	14
8	Factorial	3	2	9	0.5	14
9	Factorial	8	0	4	0.2	12
10	Factorial	8	0	4	0.5	12
11	Factorial	8	0	9	0.2	17
12	Factorial	8	0	9	0.5	17
13	Factorial	8	2	4	0.2	14
14	Factorial	8	2	4	0.5	14
15	Factorial	8	2	9	0.2	19
16	Factorial	8	2	9	0.5	19
17	Center	5.5	1	6.5	0.35	13
18	Center	5.5	1	6.5	0.35	13
19	Center	5.5	1	6.5	0.35	13

### Preparation of samples

Each of the nineteen formulas has been prepared as follows: Lactose was dissolved in pure distilled water; sodium-caseinate was added slightly under agitation at 250 rpm, and all heated in a bath at 70 °C to obtain a homogeneous solution. When starch was required, it was added once the glass beaker was removed from the water bath and cooled at the ambient temperature. The final mixture was water bath heated at 82–85 °C for 30 min to pasteurize and to allow hydration and swelling of starch granules. Samples were then cooled to 42 °C, which is the incubation temperature that is recommended for the starter culture, and inoculated with different doses of ferments. All these mixtures were poured into glass-jars and placed in a water-bath heated at 42 °C for 5–6 h. All samples were then stored at 4–5 °C for 24 h before proceeding to the evaluation of their syneresis. The analysis was carried out for 24 h. The volumes of the whey released by the fermented samples were measured by inverting each sample on fine test sieve (mesh width 0.25 mm) that was placed on top of a beaker. The volume of whey drained was collected in a graduated cylinder for each sampling time within 24 h weighed and recorded as index of syneresis (%) [25, 26].

### Experimental design set to a fixed dry matter

In this experiment, the design was adapted to a fixed dry matter. The concentration of the ferment was fixed to 0.35 g/l and the dry matter content of all samples was set at 12% with respect to the same proportions used in the experimental design 1 as showed in Table 3. The syneresis was measured as indicated bellow.

**Table 3:** Experimental design with a fixed rates of ferment and dry matter

Corresponding sample in Table 2	Sample	Sodium caseinate %(A)	Starch %(B)	Lactose %(C)	Ferment lg/l(D)	%Dry matter
1–2	I	5.142	0	6.857	0.35	12%
3–4	II	3	0	9	0.35	12%
5–6	III	4	2.66	5.33	0.35	12%
7–8	IV	2.57	1.714	7.714	0.35	12%
9–10	V	8	0	4	0.35	12%
11–12	VI	5.647	0	6.35	0.35	12%
13–14	VII	6.857	1.714	3.428	0.35	12%
15–16	VIII	5.05	1.26	5.68	0.35	12%

### pH and potential zeta monitoring

The purpose of this experiment was to evaluate the impact of starch and lactose on the acidification profile of the fermented mixed model system. According to the syneresis results of the second experimental design (Table 3), four samples were designed to be pH monitored and syneresis measured as presented in Table 4. Sodium caseinate was set at 5% and ferment at 0.35 g/l in all sample. The pH was continuously measured along the fermentation process. by immersing the glass electrode of the pH meter on samples.

**Table 4:** Formulas for pH and Zeta Monitoring

Sample	Sodium caseinate %	Starch %	Lactose %	Ferment g/l
i	5	2	9	0.35
ii	5	0	9	0.35
iii	5	2	4	0.35
iv	5	0	4	0.35

The selection of samples for zeta potential monitoring was based on the variation of the acidification profile observed in the four samples during their pH monitoring. The Samples that exhibited the shortest (sample i) and the longest (sample iv) time of gelation were selected. Zeta potential measurements were determined on dilute dispersions of each sample, the type of diluent greatly influenced the obtained results. Samples were diluted 100-fold with ultra pure distilled water before conducting the measurement while dilution with buffer solutions was unsuccessful.

### Data analysis

All statistical analyses were performed using the Design Expert version 10, a statistical software package from Stat-Ease Inc. that is specifically dedicated to performing design of experiments (DOE). In order to reduce scattering effects and to compare the samples, all physical results had been normalised. The Residual analysis, the coefficient of determination (adjusted R^2^), the significance of the models and the lack of fit were used to check the quality of the model. The robustness of the models was evaluated by determining the squared correlation coefficient (R^2^) for predicted versus measured values in cross-validation and the ratio of standard deviation (SD) to root mean square error of calibration (RMSEC) of the data sets. The ratio of the SD to the RMSEP, called the ratio of prediction to deviation (RPD), is the factor, by which the prediction accuracy has been increased compared to using the mean composition for all samples. This ratio is desired to be larger than 2 for a good calibration. An RPD ratio less than 1.5 indicates poor predictions and the model cannot be used for further prediction.

## Results and discussion

### Syneresis evaluation

Casein gels are dynamic by nature [10]. The excessive rearrangements of casein particles gel network during gelation and storage have been implicated as being responsible for whey separation and several rheological conditions of acid-induced gel network [8]. However this spontaneous process that is subsequent to the gel contraction with no external forces applied remain far from being completely controlled [27].

**Fig. 1:**
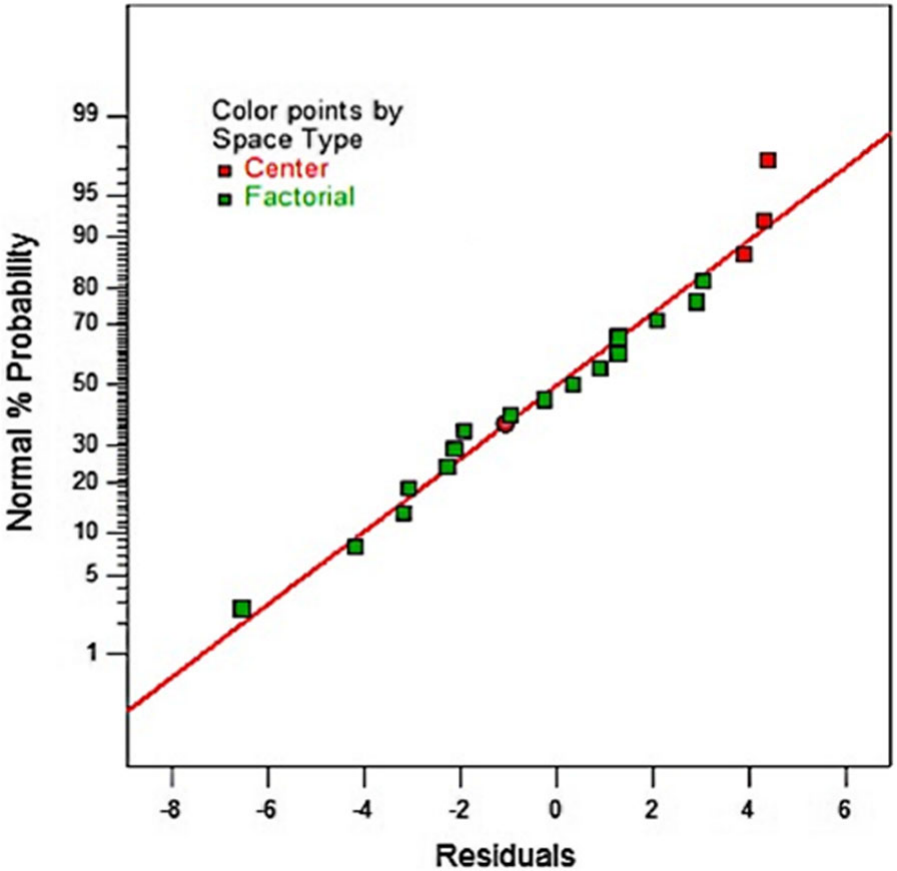
Normal Plot of Residuals

**Fig. 2:**
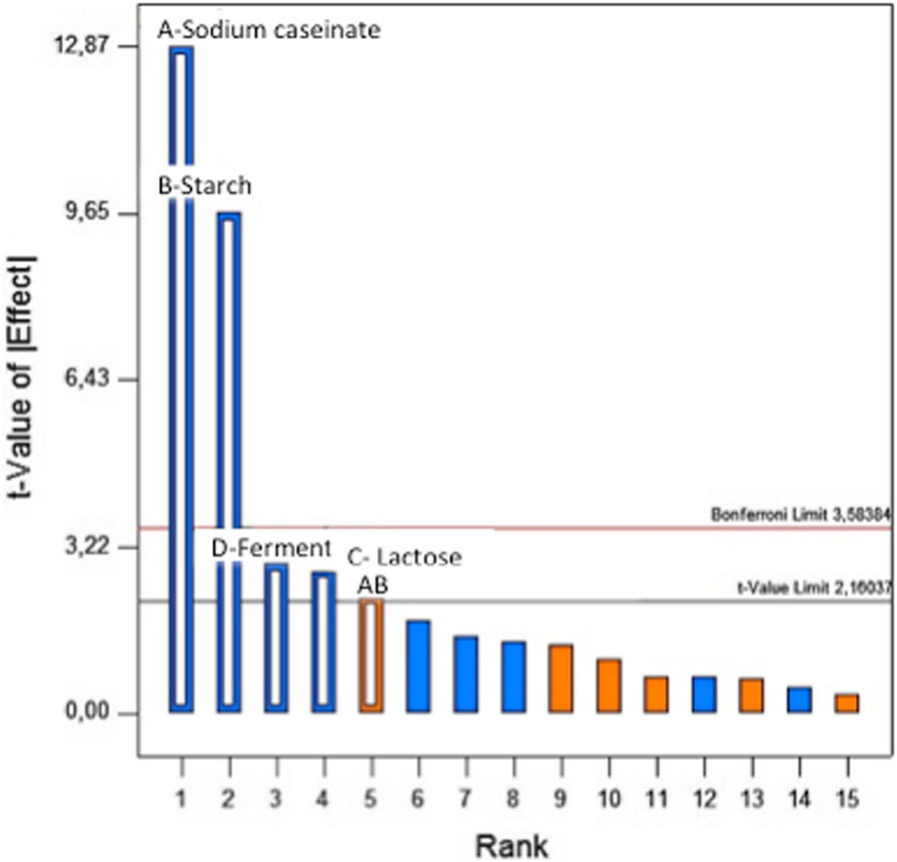
Pareto Chart

The first global experimentation with Design Expert 10 software showed a large variations ranging between a minimum of 1.8% when all factors were at their high level and a maximum of 47.8% when they were at their low level as showed in Table 5, with a “very normal distribution” of the residues (Fig. 1). The significance tests were valid and it was not necessary to transform the response to improve its modeling even if the three centered points showed a very little curvature and lack of adjustment. The Pareto Chart clearly indicated the two factors (A: Sodium caseinate and B: starch) above the Bonferroni limit (Fig. 2), showing their dominant influence on the syneresis.

**Table 5:** Syneresis results

Sample	Sodium Caseinate %(A)	Starch %(B)	Lactose %(C)	Ferment %(D)	Syneresis %	Dry Matter %
Influence of the selected factors on syneresis						
1	3	0	4	0.2	47.8	7
2	3	0	4	0.5	35.6	7
3	3	0	9	0.2	41.4	12
4	3	0	9	0.5	41	12
5	3	2	4	0.2	31.4	9
6	3	2	4	0.5	25.4	9
7	3	2	9	0.2	21.2	14
8	3	2	9	0.5	15.8	14
9	8	0	4	0.2	20.6	12
10	8	0	4	0.5	19	12
11	8	0	9	0.2	17.6	17
12	8	0	9	0.5	17.2	17
13	8	2	4	0.2	10.2	14
14	8	2	4	0.5	8.4	14
15	8	2	9	0.2	9.2	19
16	8	2	9	0.5	1.8	19
17	5.5	1	6.5	0.35	27.9	13
18	5.5	1	6.5	0.35	27.8	13
19	5.5	1	6.5	0.35	27.4	13
Syneresis after fixed rates of ferment and dry matter						
I	5.142	0	6.857	0.35	33	12
II	3	0	9	0.35	63	12
III	4	2.66	5.33	0.35	24	12
IV	2.57	1.714	7.714	0.35	48	12
V	8	0	4	0.35	5	12
VI	5.647	0	6.35	0.355	36	12
VII	6.857	1.714	3.428	0.35	12	12
VIII	5.05	1.26	5.68	0.35	29	12
Syneresis on formulas of pH and zeta potential monitoring						
i	5	2	9	0.35	28	16
ii	5	0	9	0.35	46	14
iii	5	2	4	0.35	20	11
iv	5	0	4	0.35	45	9

**Fig. 3:**
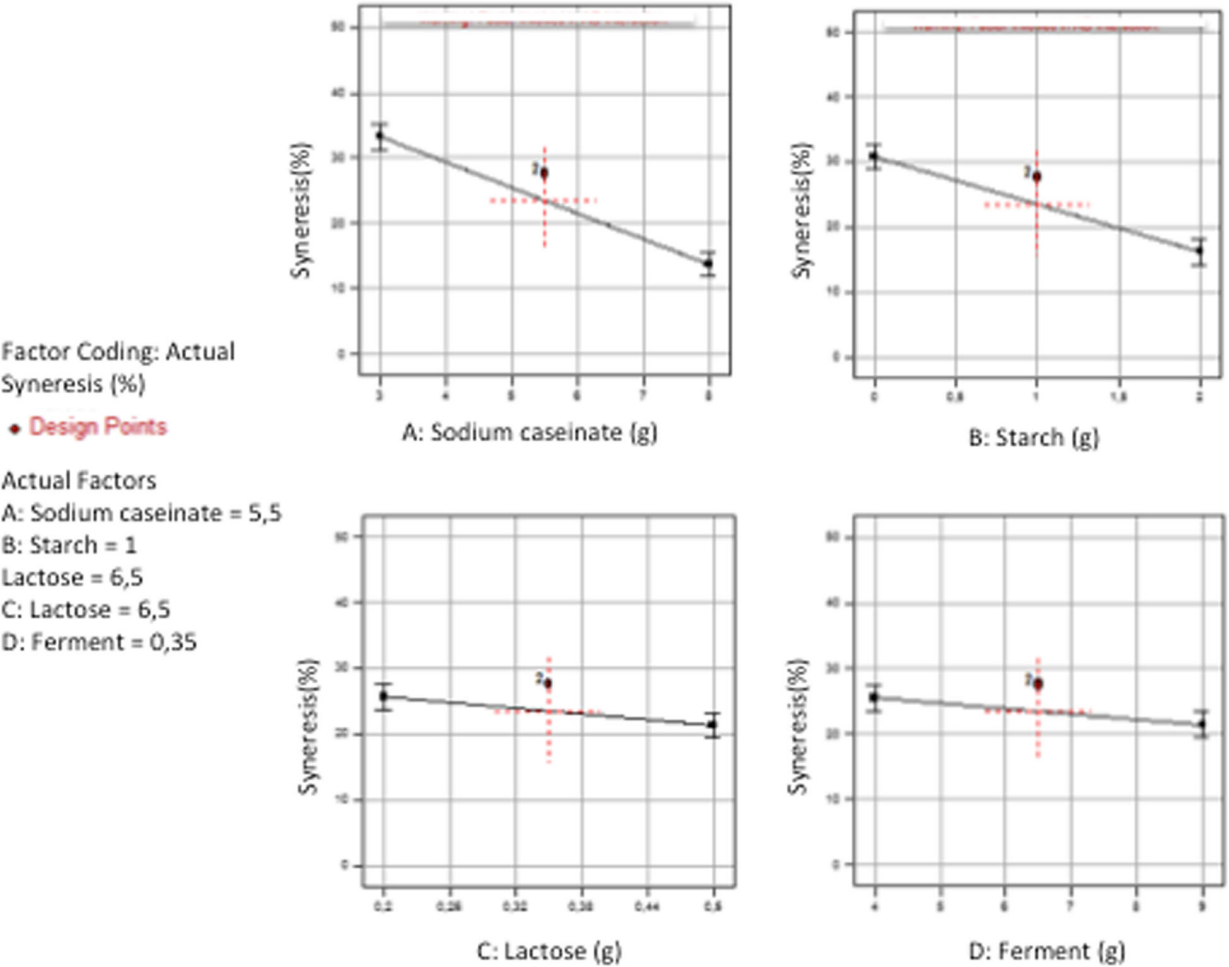
Visualization of the average negative influence of the four factors

**Fig. 4:**
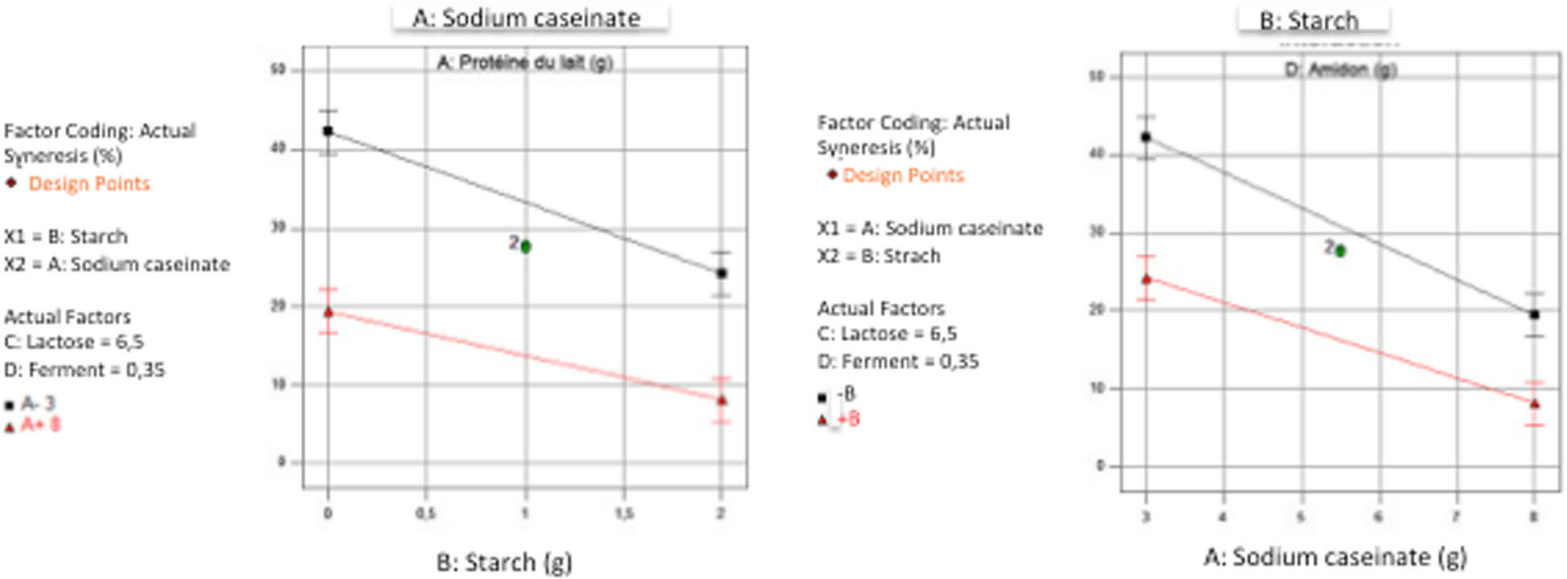
Visualization of the combined influence of factor **a** (sodium caseinate) and factor **b** (Starch) under two symmetrical angles

**Fig. 5:**
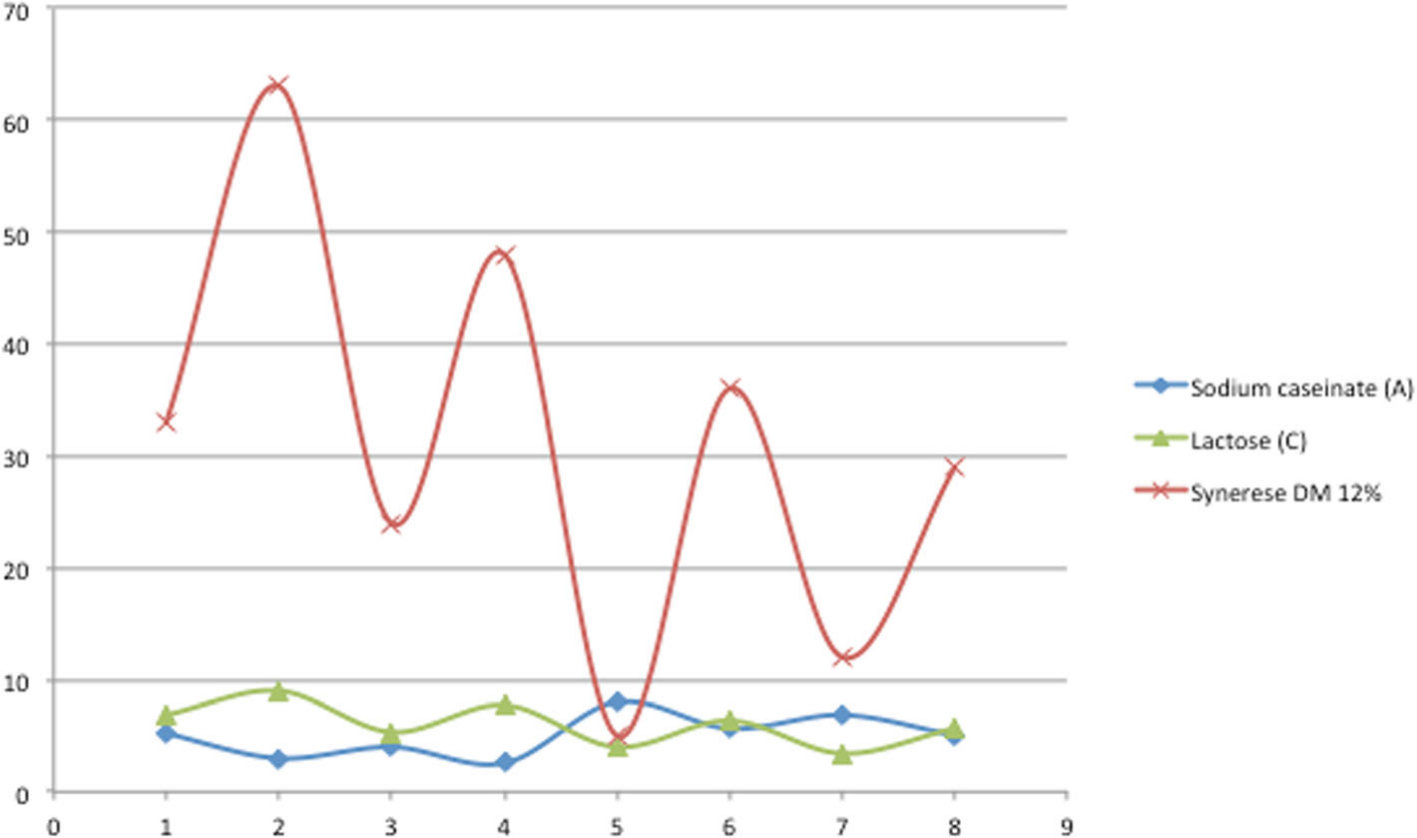
Impact of lactose and sodium caseinate on syneresis in a model system set at 12% of Dry Matter (DM)

The separate influence of the four parameters also showed the dominant influence of A & B factors (Fig. 3). The Syneresis decreased highly (19.44 and 14.60%) when the factors A (Sodium caseinate) and B (Starch) varied from their low (3 and 0) to their high level (8 and 2) while the Syneresis decreased smoothly (only 4.16 and 4.40%) when the factors C (Lactose) and D (Ferments) varied from their low level (4 and 0.2) to their high level (9 and 0.5) respectively. Furthermore, the action of the two factors whose effects were the most important (A: Sodium caseinate & B: Starch) showed a positive interaction (+ 1.70); Moreover the results revealed that when sodium caseinate and starch were combined together, sodium caseinate had more significant effect on syneresis than starch (Fig. 4). This was also confirmed by the second experimental design in which the dry matter content of all samples was fixed to 12% and syneresis measurements ranged between a minimum of 5% for the sample at 8% sodium caseinate and a maximum of 63% for the sample with 3% sodium caseinate regardless of their lactose concentrations (Fig. 5). This variation could be linked to the protein content in relation to the total solids level. Accordingly, the casein content played the major role of all solids contents in reducing syneresis, which was also observed in other previous works [28]. These results may be explained in terms of a complete denaturation of the protein at its isoelectric point showing more hydrophilic parts, which improved the water holding capacity of the resulting caseinate gel and therefore exhibited less syneresis [17]. In addition, it was reported that higher concentrations of casein originate a protein matrix less prone to whey expulsion, probably due to larger particles volume that leads to an increase of the osmotic pressure in the gel system by inter-particles repulsion, thus more resistance to gel shrinkage. This kind of stabilization can be alternatively viewed as an osmotic effect [7]. Furthermore, Kunitz in 1928 also reported that the two important factors that must play a major role in the phenomenon of syneresis, were namely the osmotic pressure and the elasticity of the polymeric gel matrix [29].

The significant impact of starch on syneresis confirms the fact that starches along with other hydrocolloids have been evaluated in yoghurt formulations as ways to achieve a desired viscosity and reduce syneresis [30]. The range of starch and milk ingredients interactions is large and the effect on the physico-chemical properties of the mixed system depends on their relative concentrations, the physico-chemical properties of starch and milk ingredients as well as the composition of milk ingredients. In these systems, Zuo et al.(2008) suggested that the main effect of starch granules is to increase the milk protein concentration during swelling by absorbing water from the continuous phase [31]. Therefore, the starch granules have to be destabilized by disrupting the native crystalline structure into an amorphous one that swells by absorbing a considerable amount of water. In fact, upon heat treatment, Hydroxyl groups in glucose monomers of amylose and amylopectin bind water and thus form a viscous solution in which the casein micelles are trapped and reduce syneresis [21]. Accordingly, the functionality of the native starch is related to the gelatinization process rather to its interaction with the caseinate aggregates [7, 22]. This results is in accordance with ours findings. Therefore, the decrease of syneresis among the studied samples could be chiefly attributed to sodium caseinate and then to starch.

### pH & zeta monitoring

**Fig. 6:**
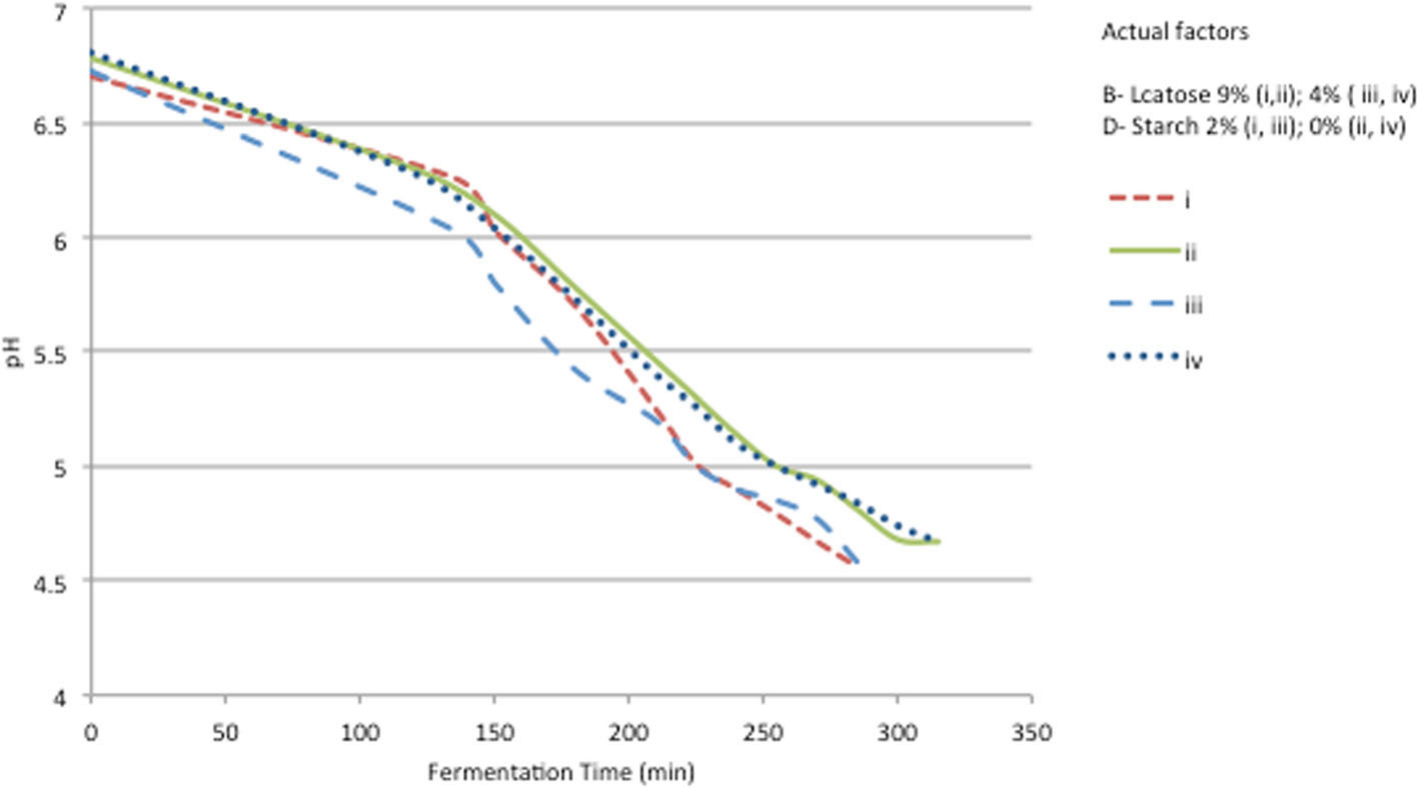
Effect of starch addition and lactose on the acidification profiles of samples set at 5% of A-sodium caseinate and 0,35 g/l of C-Ferment

To understand the effect of these formulation variables on syneresis, and their impact on the quality characteristics of the formed acid gel, a pH and zeta potential monitoring of four designed samples were conducted for controlling the fermentation process and predicting the physical properties of the final product in terms of acidification and ionic strength. Based on our results, syneresis was reduced when sodium caseinate concentration was used above 5%. Therefore, to evaluate starch and lactose impact on the acidification process all samples were designed to be set at 5% of sodium caseinate, while lactose and starch concentrations ranged between (4–9%) and (0–2%) respectively as indicated in Table 4. During fermentation process, acidification monitoring was carried out in an incubator by maintaining the temperature at 42 °C until the fermentation was complete and the pH reached 4.5–4.6. The fermentation time can be considered as the time required for the pH to decrease till end point, presupposing that the required quality properties have been developed [6]. The pH values measured at different time intervals showed that the pH decrease is slightly faster in both samples with 2% starch compared to the acid gels samples prepared without starch (Fig. 6). The difference in the incubation time was found to be significant in samples with 2% starch, 285 min comparing to 315 min in samples without starch. It can be concluded that lactose enhancement did not have any significant impact on the incubation time of samples, while the addition of starch affected slightly the acidification profile of the fermented model and therefore its fermentation time (average increase in tpH4.5 of 30 min). A similar observation was reported by Williams et al. (2003) upon the addition of starch to milk before acidification to make yogurt [32].

**Fig. 7:**
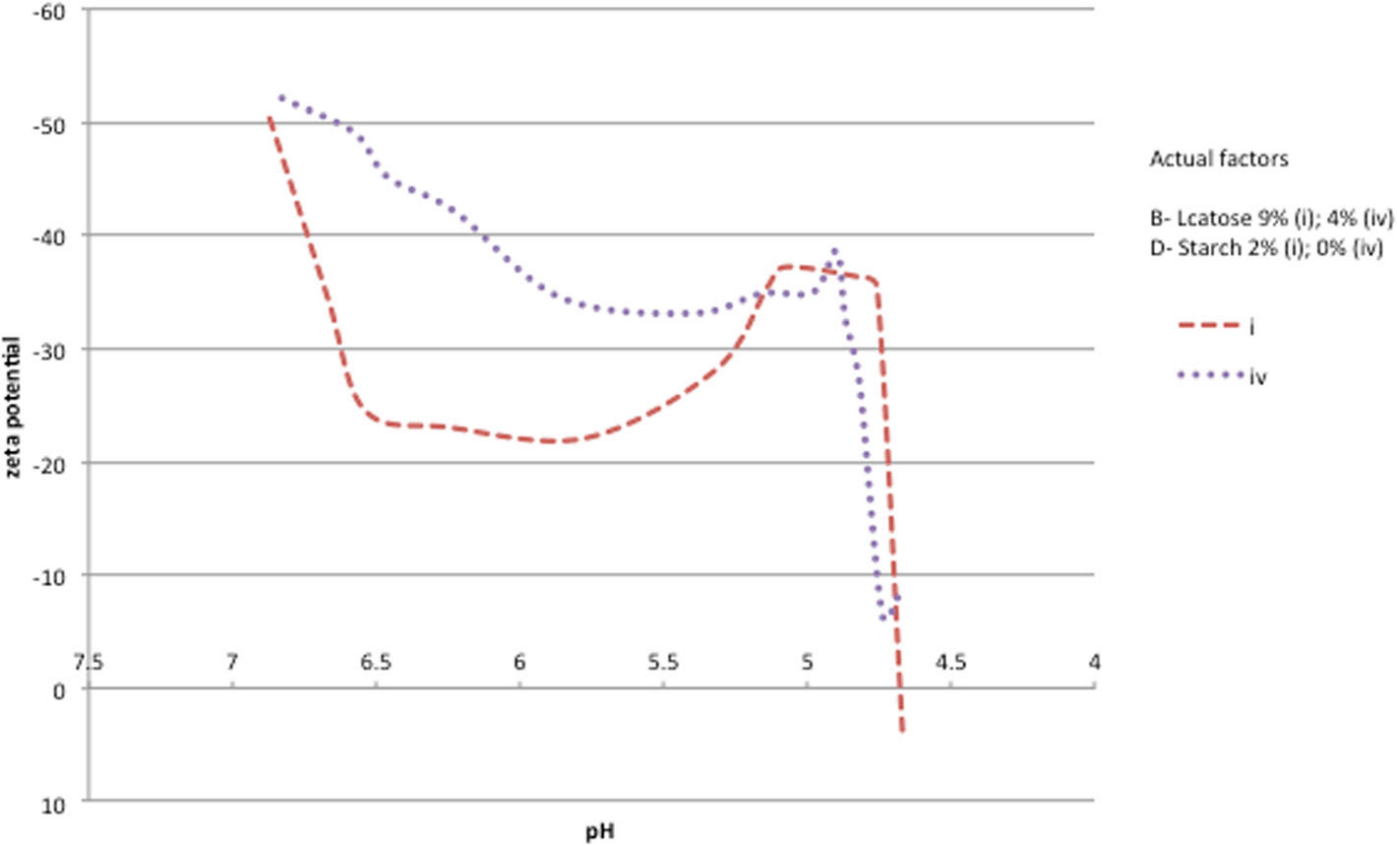
Zeta potential monitoring in samples set at 5% of A-sodium caseinate and 0,35 g/l of C-Ferment

Furthermore, the two samples showing the highest and the shortest acidification profile were zeta potential monitored. This later had been used as an indicator of the electrical charge of caseinates’ particles to measure the strength of repulsion or attraction between particles in both samples along the fermentation process. It was observed that the zeta potential decreased in both samples during acidification as showed in Fig. 7. However, a faster decrease of zeta potential was noticed in sample (i), which is probably responsible for its short fermentation time comparing to sample (iv).

In fact as the pH decreased, the proteins charges got neutralized, which minimized their electrostatic and steric forces inducing a new arrangement of micelles. Consequently, the system became unstable earlier in the sample with starch and tended to flocculation because of the less repulsive forces with the DLVO classical theory [33]. Therefore, an increase in the aggregation state of sodium caseinate and the formation of an acid gel protein network [34]. According to our results, the addition of starch shortened the incubation time, and had a detrimental effect on reducing syneresis in the fermented model, probably because of the reduction of free water and the increase of the solids contents proportion.

## Data Availability

The datasets used and/or analysed during the current study are available from the corresponding author on reasonable request.

## References

[1] Pang G, Xie J, Chen Q, Hu Z (2012). How functional foods play critical roles in human health. Food Sci Human Wellness.

[2] Valentini L, Pinto A, Bourdel-Marchasson I, Ostan R, Brigidi P, Turroni S, Hrelia S, Hrelia P, Bereswill S, Fischer A (2015). Impact of personalized diet and probiotic supplementation on inflammation, nutritional parameters and intestinal microbiota - the "RISTOMED project": randomized controlled trial in healthy older people. Clin Nutr.

[3] Marsh AJ, Hill C, Ross RP, Cotter PD (2014). Fermented beverages with health-promoting potential: past and future perspectives. Trends Food Sci Technol.

[4] Early R (2012). Dairy products and milk-based food ingredients.

[5] Akalın AS, Unal G, Dinkci N, Hayaloglu AA (2012). Microstructural, textural, and sensory characteristics of probiotic yogurts fortified with sodium calcium caseinate or whey protein concentrate. J Dairy Sci.

[6] Soukoulis C, Panagiotidis P, Koureli R, Tzia C (2007). Industrial yogurt manufacture: monitoring of fermentation process and improvement of final product quality. J Dairy Sci.

[7] Mizrahi S (2010). Syneresis in food gels and its implications for food quality.

[8] Lucey* JA (2001). The relationship between rheological parameters and whey separation in milk gels. Food Hydrocoll.

[9] Heertje I, Visser J, Smits P (1985). Structure Formation in Acid Milk Gels.

[10] Luceyub* JA, TvVb KG, Geurtsb T, Walstrab P (1997). Properties of acid casein gels made by acidification with Glucono-Nactone. 2. Syneresis, permeability and microstructural properties. Int Dairy J.

[11] O’Kennedy BT, Mounsey JS, Murphy F, Duggan E, Kelly PM (2006). Factors affecting the acid gelation of sodium caseinate. Int Dairy J.

[12] Manab* A (2017). Casein polysaccaharides interaction – a review. Int J ChemTech Res.

[13] Sadeghi M, Madadlou A, Khosrowshahi A, Mohammadifar M (2014). Acid-induced gelation behavior of casein/whey protein solutions assessed by oscillatory rheology. J Food Sci Technol.

[14] Horne DS (1998). Casein interactions: casting light on theBlackBoxes, the structure in dairy products. Int Dairy J.

[15] Lucey jA. Perspectives on casein interactions. Int Dairy J:56–65.

[16] S.P.F.M Roefs TVV: Structure of acid casein gels 2. Dynamic measurements and type of interaction forces colloids and surfaces 50:161–175.

[17] Lapointe-Vignola C. Fondation de technologie laitière du Q: Science et technologie du lait: transformation du lait: Presses internationales Polytechnique; 2002.

[18] Guo C, Campbell BE, Chen K, Lenhoff AM, Velev OD (2003). Casein precipitation equilibria in the presence of calcium ions and phosphates. Colloids Surf B: Biointerfaces.

[19] Considine T, Noisuwan A, Hemar Y, Wilkinson B, Bronlund J, Kasapis S (2011). Rheological investigations of the interactions between starch and milk proteins in model dairy systems: a review. Food Hydrocoll.

[20] Lobato-Calleros C, Ramírez-Santiago C, Vernon-Carter EJ, Alvarez-Ramirez J (2014). Impact of native and chemically modified starches addition as fat replacers in the viscoelasticity of reduced-fat stirred yogurt. J Food Eng.

[21] Gyawali R, Ibrahim SA (2016). Effects of hydrocolloids and processing conditions on acid whey production with reference to Greek yogurt. Trends Food Sci Technol.

[22] N-x S, Liang Y, Yu B, C-p T, Cui B (2016). Interaction of starch and casein. Food Hydrocoll.

[23] Bourlioux P, Braesco V, Mater DDG (2011). Yaourts et autres laits fermentés. Cahiers de Nutrition et de Diététique.

[24] Tamime A.Y., Robinson R.K. (2007). Biochemistry of fermentation. Tamime and Robinson's Yoghurt.

[25] Kneifel W, Paquin P, Abert T, Richard JP (1991). Water-holding capacity of proteins with special regard to Milk proteins and methodological aspects—a review. J Dairy Sci.

[26] Cueva O, Aryana KJ (2008). Quality attributes of a heart healthy yogurt. LWT Food Sci Technol.

[27] Lucey JA (2002). Formation and physical properties of Milk protein gels. J Dairy Sci.

[28] Fiszman SM, Lluch MA, Salvador A (1999). Effect of addition of gelatin on microstructure of acidic milk gels and yoghurt and on their rheological properties. Int Dairy J.

[29] Kunitz M (1928). Syneresis and swelling of gelatin. The Journal of General Physiology.

[30] Keogh MK, O’Kennedy BT (1998). Rheology of stirred yogurt as affected by added Milk fat, protein and hydrocolloids. J Food Sci.

[31] Zuo JY, Hemar Y, Hewitt S, Saunders A (2008). Effect of the extent of pasting on the dynamic rheological properties of acidified skim milk gels containing normal rice starch. Food Hydrocoll.

[32] Williams R, Glagovskaia O, Augustin MA (2003). Properties of stirred yogurts with added starch: effects of alterations in fermentation conditions. Aust J Dairy Technol.

[33] Tholstrup Sejersen M, Salomonsen T, Ipsen R, Clark R, Rolin C, Balling Engelsen S (2007). Zeta potential of pectin-stabilised casein aggregates in acidified milk drinks. Int Dairy J.

[34] Lucey WJLJA. Structure and physical properties of yogurt gels: effect of inoculation rate and incubation temperature. American Dairy Science Association. 2004. 10.3168/jds.S0022-0302(04)73450-5 15377593

